# Solvent-Engineered PEACl Passivation: A Pathway to 24.27% Efficiency and Industrially Scalable Perovskite Solar Cells

**DOI:** 10.3390/nano15090699

**Published:** 2025-05-06

**Authors:** Min Xin, Ihtesham Ghani, Yu Zhang, Huaxi Gao, Danish Khan, Xin Yang, Zeguo Tang

**Affiliations:** 1 School of Energy and Environmental Sciences, Yunnan Normal University, Juxian Road 768, Chenggong, Kunming 650500, China; 2223160035@ynnu.edu.cn (M.X.); 2223160030@ynnu.edu.cn (H.G.); 2 The College of New Materials and New Energies, Shenzhen Technology University, Lantian Road 3002, Pingshan, Shenzhen 518118, China; 2351416001@email.szu.edu.cn (I.G.); khandanish@sztu.edu.cn (D.K.)

**Keywords:** perovskite film quality, PEACl additive, solvent engineering, high-performance PSCs

## Abstract

Addressing the critical challenges of interfacial defects and insufficient stability in perovskite solar cells, this work introduces a co-solvent engineering strategy to dynamically regulate the phenethylammonium chloride (PEACl) passivation layer. The effect of isopropyl alcohol (IPA) and a DMSO: IPA (1:100) mixture as solvent for forming the PEACl 2D passivation layer is systematically explored, and the synergistic interplay between solvent coordination strength and crystallization kinetics is systematically investigated. The DMSO: IPA (1:100) blend balances Pb-O coordination (via DMSO) and rapid phase separation (via IPA), enabling the oriented growth of a dense, ultrathin 2D perovskite overlayer. This suppresses defect density (electron traps reduced to 1.68 × 10^15^ cm^−3^) and extends carrier lifetime, yielding a champion power conversion efficiency (PCE) of 24.27%—a significant improvement over the control (22.73%). For the first time, we establish a dual-parameter “solvent coordination-crystallization kinetics” model, providing a universal framework for designing environmentally benign solvent systems and advancing the industrial scalability of high-performance perovskite solar cells (PSCs).

## 1. Introduction

Organic–inorganic hybrid perovskite solar cells (PSCs) have achieved certified power conversion efficiencies (PCEs) exceeding 26%, rivaling silicon photovoltaics, and offer advantages in solution processability and tunable optoelectronic properties [1,2]. In recent years, inverted (p-i-n) perovskite solar cells have demonstrated superior power conversion efficiencies compared to their conventional (n-i-p) counterparts [3]. P-i-n PSCs have long garnered substantial research interest owing to their reduced hysteresis effects and compatibility with simplified fabrication processes [4,5]. However, their limited device performance, particularly their poor operational stability, poses critical challenges for practical deployment. Since 2016, significant advancements in both the power conversion efficiency (PCE) and operational stability of p-i-n PSC have been achieved, driven by innovations in defect passivation, interface engineering, solvent engineering, morphological control, and charge transport layer (CTL) design [6,7]. Consequently, certified efficiency records for this architecture have been continuously updated. P-i-n perovskite solar cells demonstrate compelling advantages for monolithic perovskite–silicon tandem solar cells and large-scale deployment scenarios [8]. However, interfacial defect states at perovskite surfaces and grain boundaries (GBs) remain a critical bottleneck, inducing non-radiative recombination, ion migration, and accelerated degradation under operational stressors [9,10,11,12]. Moreover, charge recombination at interfaces and GBs significantly impairs the efficiency of charge extraction, thereby adversely affecting device performance metrics, such as short-circuit current (J_SC_) and fill factor (FF), through increased series resistance. Extensive research has established a direct correlation between trap density, mobile ionic defect concentration, and the magnitude of J–V hysteresis, whereby the presence of defect states or migratory ions not only limits carrier transport efficiency but also exacerbates hysteresis behavior in PSCs [13]. These defects, primarily undercoordinated Pb^2+^, halide vacancies, and metallic Pb^0^ clusters, act as Shockley–Read–Hall recombination centers, reducing the open-circuit voltage (V_OC_) and fill factor (FF). Furthermore, defect-mediated ion migration accelerates hysteresis and phase segregation, thereby undermining long-term stability [14]. Defect passivation strategies can alter contact resistance at the interface between the perovskite and charge transport layers. Additionally, the addition of passivation agents to perovskite precursors can change film morphology, reducing the presence of pinholes as shunt paths, thus affecting the FF in PSCs. To mitigate these issues, 2D/3D heterojunctions formed via phenylammonium ion passivation have emerged as a promising strategy. The in situ growth of a wide-bandgap 2D perovskite overlayer suppresses interfacial recombination and enhances moisture resistance [15,16,17].

Chen et al. reported a rationally designed 2D/3D perovskite stacked layer, which greatly improved the stability of PSCs by growing a 2D PEA_2_PbI_4_ capping layer on a 3D perovskite film [18]. Subsequently, in another well-known work, phenylethylammonium (PEA)-based 3D/2D heterojunctions were formed by adding lead overdose to the original perovskite film, and the PEAI was spin coated, resulting in a more uniform 2D/3D heterojunction [19]. Furthermore, a dual-nature passivation strategy for p-i-n PSCs was proposed, using a long-chain alkyl ammonium salt (PEACl) to simultaneously passivate defects at the perovskite/C60 interface as well as in grain boundaries, achieving significant improvements in V_OC_ and FF [20]. Although changing the cation design helps in promoting the charge transfer and stability of 3D/2D perovskite films, discovering the solvent role will be more interesting. As IPA is detrimental to the 3D perovskite film, it has been demonstrated that adding DMF solvent could promote PEAI to penetrate into perovskites and form an optimal 3D/2D perovskite heterostructure, which is beneficial for passivating the trap states and enhancing charge transport [21]. Li and coworkers utilized chlorobenzene (CB) as a diluent to reduce the amount of IPA and suppress defects caused by additional solvents. They discovered a ternary solvent system that shows good applicability and reliability in different passivating agents [22]. Although CB is compatible with passivators, it suffers from rapid volatilization-induced inhomogeneity and poor control over PEA molecular orientation, limiting both efficiency and stability [23]. DMF is mainly used as a high-solubility solvent, and its weak coordination ability forms a complex (PbI_2_·DMF) with poor stability, which requires higher concentrations to effectively dissolve PbI_2_, making it prone to local supersaturation due to volatilization and resulting in inhomogeneous crystallization. In contrast, DMSO forms a stable coordination intermediate (PbI_2_·DMSO), delaying the crystallization process, promoting the growth of large grains, and improving the uniformity of the film [24].

Here, we propose a solvent co-engineering paradigm that decouples coordination and volatilization kinetics to dynamically regulate PEACl passivation. By employing a DMSO: IPA (1:100) mixed solvent, we exploit DMSO’s strong Pb-O coordination to align PEA^+^ along the (001) perovskite plane, while IPA’s rapid phase separation confines 2D perovskite growth to an ultrathin, pinhole-free morphology. This dual-parameter approach addresses the longstanding trade-off between defect passivation completeness and interfacial charge transport efficiency [25,26]. Our work advances perovskite photovoltaics in three key dimensions:

(1) We establish a universal “solvent coordination-volatilization kinetics” model, experimentally validated through in situ XRD and XPS, that links solvent properties to passivation layer quality.
(2) DMSO/IPA-engineered devices achieve a PCE of 24.27% (V_OC_ = 1.15 V) with 90% efficiency retention after 1344 h in inert atmospheres, outperforming single-solvent control systems.
(3) By replacing toxic chlorobenzene with a low-DMSO green solvent system, this strategy aligns with industrial requirements for scalable, eco-friendly manufacturing.

## 2. Experimental Section

### 2.1. Materials

All chemical reagents are directly dissolved without additional purification steps. Chlorobenzene (CB, anhydrous, 99.8%), N, N-dimethylformamide (DMF, anhydrous, 99.8%), bathocuproine (BCP, 96.0%), IPA, PEACl, and DMSO (anhydrous, ≥99.9%) were purchased from Sigma-Aldrich (Burlington, MA, USA). Bathocuproine (BCP, 96.0%), and NiO_X_ nanoparticles were procured from Advanced Election Technology Co., Ltd. (Yingkou, China). Lead iodide (PbI_2_, 99.999%), formamidinium iodide (FAI, 99.99%), C_60_, cesium iodide (CsI, 99.9%), methylammonium iodide (MAI, 99.99%), and methylamine hydrochloride (MACl, 99.99%) were purchased from Xi’an Shuoyuan Optoelectronic Technology Co., Ltd. (Xi’an, China). 4-(3,6-Dimethyl-9H-carbazol-9-yl) butyl] phosphonic Acid was obtained from TCI (Shanghai, China).

### 2.2. Device Fabrication

**Figure 1:**
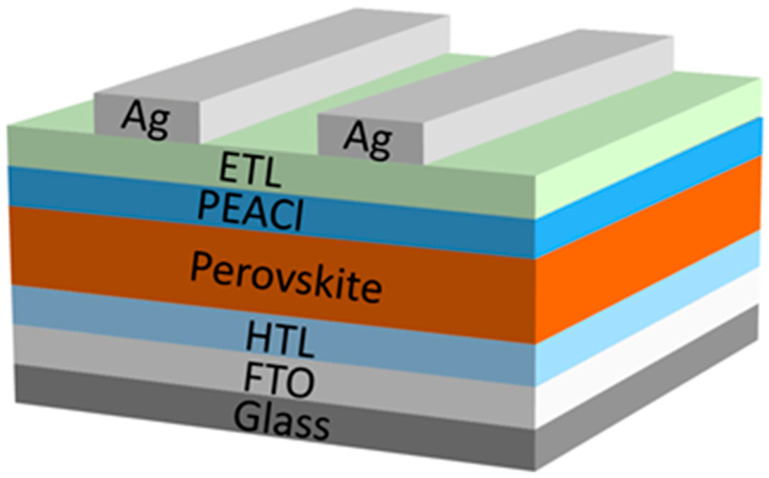
Schematic of the device structure of fabricated PSCs.

All devices were fabricated on etched FTO conductive glass (2.45 × 2.45 cm, 8 Ω/sq). Firstly, FTO was cleaned by ultrasonic cleaning in deionized water, isopropanol, and ethanol for 25 min, respectively. The surface residual solution was blown away with nitrogen and then treated with UV–ozone for 15 min to remove any chemical residues and enhance the surface wettability of FTO. NiO_X_ was dispersed in deionized water at a concentration of 20 mg·mL^−1^, and then the coating was rotated on the FTO substrate at 2000 rpm for 30 s and annealed at 150 °C for 30 min on a heating stage in an air atmosphere. After cooling, it was transferred to a glove box filled with nitrogen. Next, the SAM layer was prepared on the NiO_X_. Me-4PACz was dissolved in ethanol at a concentration of 0.5 mg·mL^−1^, spin-coated at 3000 rpm for 30 s, and then annealed at 100 °C for 10 min on a heating stage in a N_2_ atmosphere. Next, the perovskite photoactive layer was prepared. To dissolve 219.3 mg FAI, 23.8 mg MAI, 19.5 mg CsI, 12.66 mg MACl, and 760.7 mg PbI_2_ in 1 mL of mixed solvent (DMF and DMSO volume ratio of 4:1), a 1.5 M precursor solution composed of FA_0.85_MA_0.1_Cs_0.05_PbI_3_ was used. Then, the perovskite precursor solution was continuously rotated on the substrate at 1000 rpm for 10 s and 5000 rpm for 40 s. At 45 s (5 s before the end of the rotating coating), 160 μL of CB anti-solvent was rapidly dropped. Then, the film was annealed at 100 °C for 30 min on a heating stage in the N_2_ atmosphere glove box, and the surface treatment was carried out after cooling. For PEACl-treated devices, PEACl was dissolved in IPA and DMSO: IPA (1:100) at concentrations of 1 mg·mL^−1^ and spin-coated at 4000 rpm for 30 s, followed by annealing at 100 °C for 5 min. Finally, the electron transport layer and metal electrode were prepared using the thermal evaporation method under a high vacuum (<1 × 10^−4^ Pa). For the electron transport layer, 25 nm C60 and 6 nm BCP were evaporated at a rate of 0.5 Å·s^−1^ and 0.3 Å·s^−1^, respectively. The silver electrode was used as the metal electrode, and 100 nm was evaporated at a rate of 1 Å·s^−1^. The device structure is shown in Figure 1.

### 2.3. Characterization and Test

Film characterization: SEM images of perovskite films were obtained using the Gemini SEM 300 from Carl Zeiss Microscopy Ltd. (Cambridge, UK) with software SmartSEM V06.01 version. During the measurement, an acceleration voltage of 15 kV was applied when the vacuum was up to 5 × 10^−4^ mbar to obtain surface SEM images using the in-lens mode. AFM images were acquired using a Cypher S microscope from Oxford Instruments Asylum Research, Inc. (Santa Barbara, CA, USA) with the latest version of Ergo software (https://afm.oxinst.com/products/afm-software/ergo-afm-software-for-cypher-and-jupiter). The acquired image data was analyzed with the software NanoScope Analysis 3.0. The probe was RTESPA-150, a reflective aluminum-coated tip with a frequency of ≈150 kHz and a nominal tip radius of ≈8 nm. X-ray diffraction (XRD) data were collected in reflection mode at room temperature on a Rigaku (Tokyo, Japan) SmartLab diffractometer equipped with a 2D detector HyPix-400, using monochromated Cu-Kα (λ = 1.5405 Å) radiation, and Smartlab Studio II software. The scans (step size 0.01–0.02°, dwell time 0.05–0.1 s/step) were measured in a parallel beam geometry with a height-limiting slit of 0.2 mm. Optical absorption spectra were measured in the wavelength range of 500–850 nm using the Lambda 1050+ UV-Vis spectrophotometer from PerkinElmer Inc. (Waltham, MA, USA) and the software UV WinLaB 6.4.0. XPS measurements were performed on a Thermo Fisher ESCALAB 250Xi instrument with a monochromatic Mg Kα (1.254 keV) X-ray source, and the samples were measured under an ultrahigh vacuum (<10^−7^ Pa). Charge compensation was performed using electrons ionized from argon gas. UPS spectra were measured using an ESCALAB 250Xi from Thermo Fisher Scientific (Waltham, MA, USA) with a He Iα photon source (21.22 eV) and a voltage bias of −10 V was applied between the sample and the detector. Binding energy scale calibration of the UPS spectrometer was performed using a clean gold film. The results of the XPS and UPS measurements were analyzed using the software Avantage 5.976. PL and TRPL spectra were recorded using the FLS1000 spectrometer from Edinburgh Instruments Ltd. (Livingston, UK) and analyzed using the software Fluoracle 2.13.1. The PL emission peak wavelength mapping image was calculated using a 405 nm pulsed laser and the PL intensity mapping image at different emission wavelengths from 700 nm to 850 nm in steps of 10 nm. The charge carrier lifetime was obtained by fitting TRPL results through the following formula: y = y_0_ + A_1_exp$\left(\frac{-t}{\tau_{1}}\right)$ + A_2_exp$\left(\frac{-t}{\tau_{2}}\right)$. The average charge lifetime was calculated through τ_ave_ = $\frac{\sum A_{i}\tau_{i}^{2}}{\sum A_{i}\tau_{i}}$. For all tests, the concentration of PEACl used was 1 mg·mL^−1^.

Device characterization: Electrochemical impedance spectroscopy (EIS) was performed with an electrochemical workstation VersaSTAT 3 from AMETEK, Inc. (Berwyn, PA, USA) and analyzed in software VersaStudio 2.6. EQE spectra of PSCs were obtained with QE-R3011 from Enli Technology Co., Ltd. (Kaohsiung, Taiwan), using Czerny–Turner monochromatic incident light with a chopper frequency of 210 Hz. The PCE and SCLC of perovskite solar cells were calculated by recording the current density–voltage (*J-V*) curves on a solar simulator connected to a Keithley 2400 Digital Source Meter under AM1.5G irradiation in the dark. The solar simulator and testing software IVS-KA6000 are from Enli Technology Co., Ltd. (Kaohsiung, Taiwan). The Keithley 2400 Digital Source Meter is produced by Aigtek (Xian, China). A metal mask defined the active area with a size of 0.07 cm^2^. For the stability test, unencapsulated devices were stored in a nitrogen (N_2_) environment or in an environment with a humidity of 30–40%. They were periodically taken out for measurements. After the measurements were completed, they were returned to their respective storage environments to be preserved for subsequent measurements.

## 3. Results and Discussion

### 3.1. The Influence of PEACl on Perovskite Films in Different Solvents

**Figure 2:**
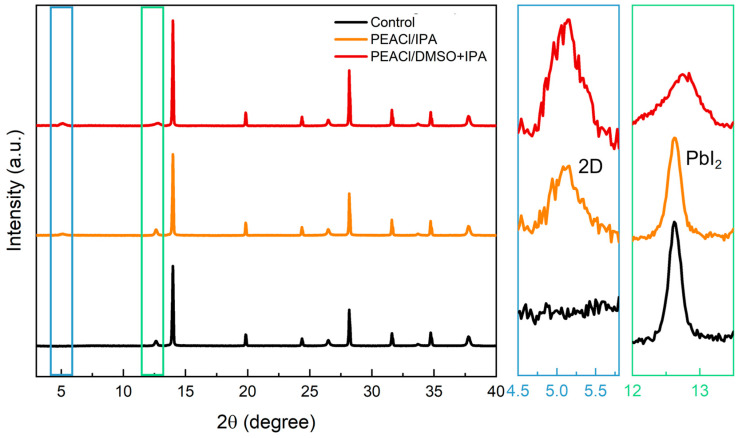
XRDs of control films and the films passivated with PEACl. Right panel is the zoomed-in version showing the peaks of low-dimensional perovskites.

To systematically investigate the modulatory effects of PEACl on perovskite film morphology and charge carrier transport in different solvent systems, we selected two different solvents to dissolve PEACl: namely the IPA solvent and the mixed solvent of IPA and DMSO. In order to understand their influence on the crystallographic orientation of perovskites, we carried out X-ray diffraction (XRD) tests. As shown in Figure 2, owing to the appearance of the 2D perovskite, the intensity of the characteristic peak of PbI_2_ decreased. The characteristic peak of the (001) crystal plane of perovskite at 13.96° gradually increased, indicating the high-quality growth of perovskite in the (001) plane orientation, and a characteristic peak belonging to the two-dimensional perovskite appeared on top of the perovskite [27,28]. However, when the solvent was only IPA, all the characteristic peaks of the α-phase perovskite were weakened. This may be because pure IPA cannot enable the residual PbI_2_ on the surface to react sufficiently with PEACl, which is not conducive to further improving device performance. This is consistent with the results of the enlarged view of the characteristic peak of PbI_2_ in the range of 12–13.4° on the left. The characteristic peak of the remaining PbI_2_ in the pure IPA solvent was stronger than that in the mixed solvent of IPA and DMSO, which greatly reduced the risk of the decrease in efficiency and stability caused by the formation of Pb^0^ [29].

**Figure 3:**
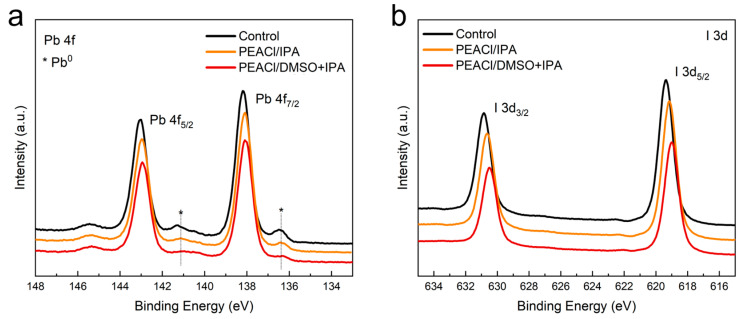
(**a**) Pb 4f and (**b**) I 3d XPS spectra.

In addition, X-ray photoelectron spectroscopy (XPS) measurements were carried out in Figure 3 to explore the interaction between PEACl and the three-dimensional perovskite film. Compared to the control film, in the film treated with PEACl in the mixed solvent of IPA and DMSO, the peaks of Pb 4f and I 3d both shifted significantly towards lower binding energies, which indicates that the chemical bonds of iodine and lead atoms in the perovskite film changed [30]. As is well known, PbI_2_ is considered to be the main source of Pb^0^, and Pb^0^ is a recombination center that has an extremely negative impact on the performance and long-term stability of the device. Pb^0^ leads to nonradiative recombination losses, which are harmful to the overall performance of the device. Because there is less residual lead iodide in the film after PEACl treatment, the generation of Pb^0^ is significantly suppressed [31]. In contrast, obvious metallic Pb^0^ peaks appeared in the control film at approximately 141 eV and 137 eV.

**Figure 4:**
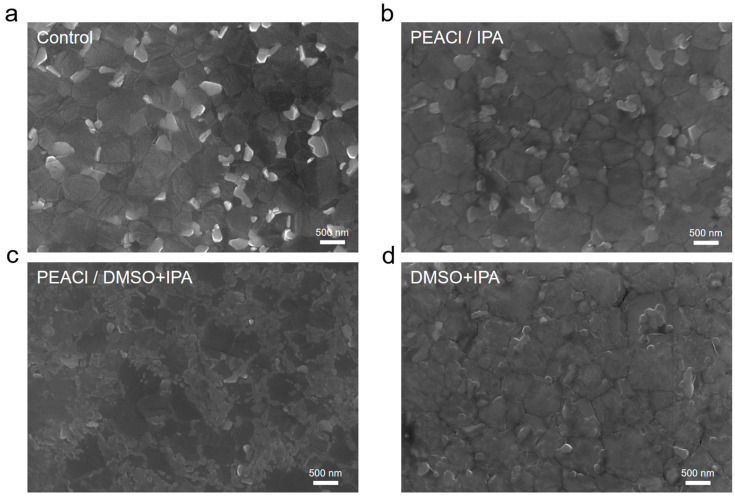
SEM images of (**a**) control film and the films treated with (**b**) PEACl/IPA, (**c**) PEACl/DMSO + IPA, and (**d**) DMSO + IPA.

It can be clearly seen from the SEM characterization results shown in Figure 4 that the perovskite films passivated with a single-solvent IPA exhibited only slight changes at the interface and grain boundaries compared to the control group. Notably, the film exhibited significant changes under mixed-solvent passivation. Crystalline grains cover the surface of the perovskite, which reduces the appearance of grain boundaries. Grain boundaries often act as the starting point for film degradation and affect the properties of thin films [32]. The interaction of DMSO with the 3D perovskite layers was further confirmed. We studied the morphology of mixed solvent (PEACl-free)-treated films. Due to the strong coordination effect of DMSO on Pb^2+^, as shown in Figure 4d, it can be seen that DMSO partially dissolves the perovskite surface, which provides a site for the passivation of PEACl. However, treatment with the solvent alone did not produce a continuous coverage of small grains, similar to that observed in Figure 4c. This suggests that the small grains in Figure 4c arise owing to the synergistic interaction between DMSO and PEACl.

**Figure 5:**
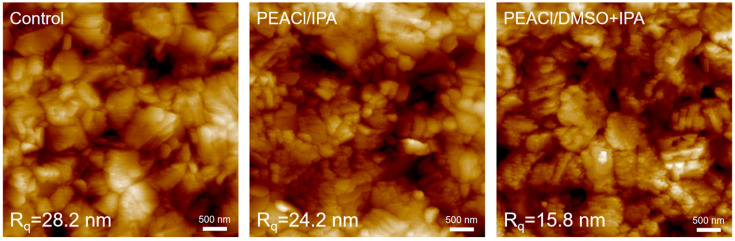
AFM images for control films and the films passivated with PEACl.

In the AFM test (Figure 5), the morphology of the perovskite film exhibited different degrees of uniformity after being passivated by different solvents. This process could help lower root-mean-square roughness (Rq) values. The Rq values for the control film and the films based on IPA and DMSO + IPA were 28.2 nm, 24.2 nm, and 15.8 nm, respectively. A smoother perovskite surface with a more uniform surface potential distribution is beneficial for forming efficient contact with the adjacent layer, which prevents nonradiative recombination.

**Figure 6:**
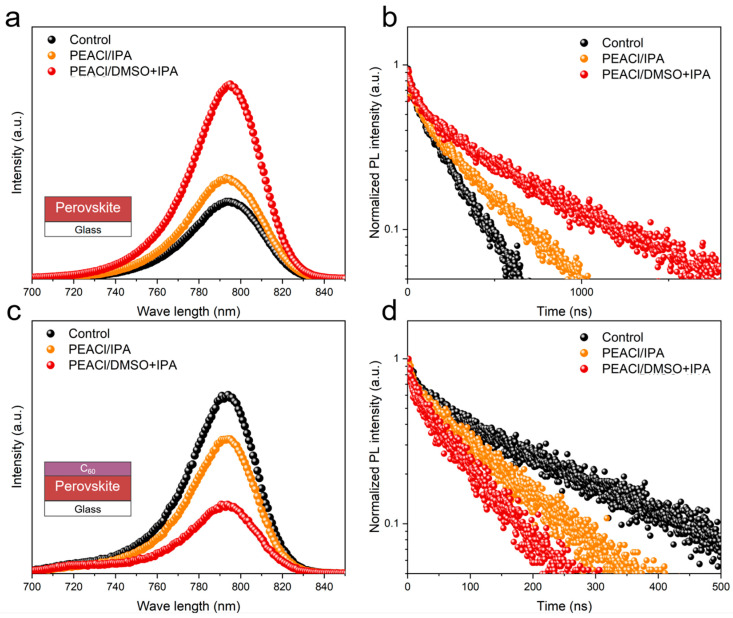
(**a**) PL intensity and (**b**) TRPL of control and target films on glass. (**c**) PL intensity and (**d**) TRPL of control and target films (with C60) on glass.

To further reveal the charge transfer and recombination behavior on the perovskite surface, photoluminescence (PL) spectroscopy was employed to characterize the perovskite films with and without the hole extraction layer. When the excitation light was incident from the perovskite side, the PL emission intensity of the film post-treated with one-dimensional perovskite increased significantly (Figure 6a). After depositing the C_60_ layer (Figure 6c), the PL intensity of the samples treated with PEACl showed obvious fluorescence quenching, indicating that the passivation effect of one-dimensional perovskite accelerated the extraction and transport of charge carriers [33]. To study the charge carrier dynamics, time-resolved photoluminescence (TRPL) measurements were also conducted for cases with and without the C_60_ layer (Figure 6b,d). The perovskite film treated with PEACl had the longest PL lifetime, whereas it had the shortest PL lifetime after depositing the C_60_ layer [34]. This suggests that PEACl can effectively passivate the surface and bulk defects, thereby reducing non-radiative recombination in the perovskite film. Moreover, the performance of the binary solvent was significantly better than that of the single solvent, indicating a better defect passivation effect.

**Figure 7:**
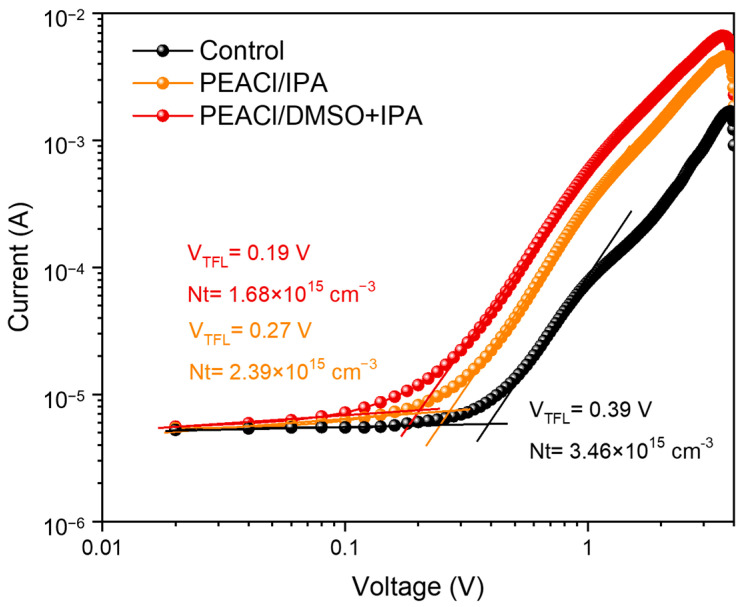
The SCLC of electron-only (FTO/ETL/perovskite/C_60_/BCP/Ag) devices under dark.

Next, the space-charge-limited current (SCLC) was determined using the structure of FTO/ETL/perovskite/C_60_/Ag to verify the electron trap density of the different perovskite films (Figure 7). Compared with the control film, after treatment with PEACl, the electron trap density decreased significantly from 3.46 × 10^15^ cm^−3^ to 2.39 × 10^15^ cm^−3^ and 1.68 × 10^15^ cm^−3^, and PEACl in the mixed solvent of DMSO and IPA exhibited a lower trap density. These results indicate that treatment with the binary solvent can significantly reduce defects in the perovskite film, such as excessive lead iodide and metallic Pb^0^, which is beneficial for the realization of efficient and stable perovskite devices [35].

**Figure 8:**
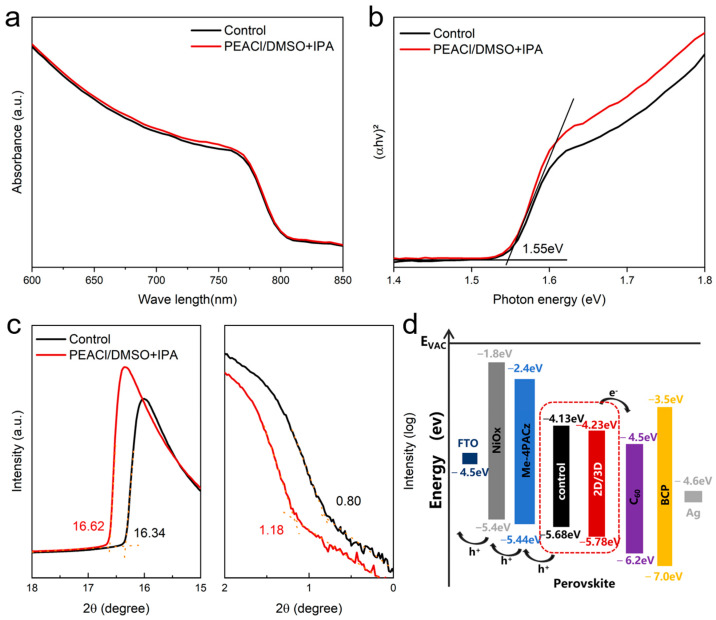
(**a**) UV–vis absorption spectra and (**b**) the corresponding Tauc analyses of control and PEACl-passivated perovskites. (**c**) UPS spectra of control and PEACl-passivated perovskites. (**d**) Band energy diagram of control and PEACl-passivated perovskites.

In addition, the optical properties of perovskite films before and after passivation were evaluated using UV–vis spectroscopy. Figure 8a shows that the absorbance of the PEACl-passivated and control films overlapped. From the UV–vis data, we obtained a Tauc plot showing that PEACl passivation did not alter the bandgap of the perovskite. The effects of PEACl passivation on perovskite surfaces were explored in detail by ultraviolet photoelectron spectroscopy (UPS), and the secondary electron cutoff (SECO) and VBM spectra are shown in Figure 8c. The surface work function of the PEACl-passivated film decreased from 4.88 eV to 4.60 eV, due to charge redistribution caused by strong surface reaction. The corresponding VBM position was moved from 0.79 eV to 1.10 eV. Moreover, it is clear that the perovskites with PEACl passivation exhibited a better band alignment, which promotes efficient charge transfer to the transport layers.

### 3.2. Performance of PSC Devices

**Figure 9:**
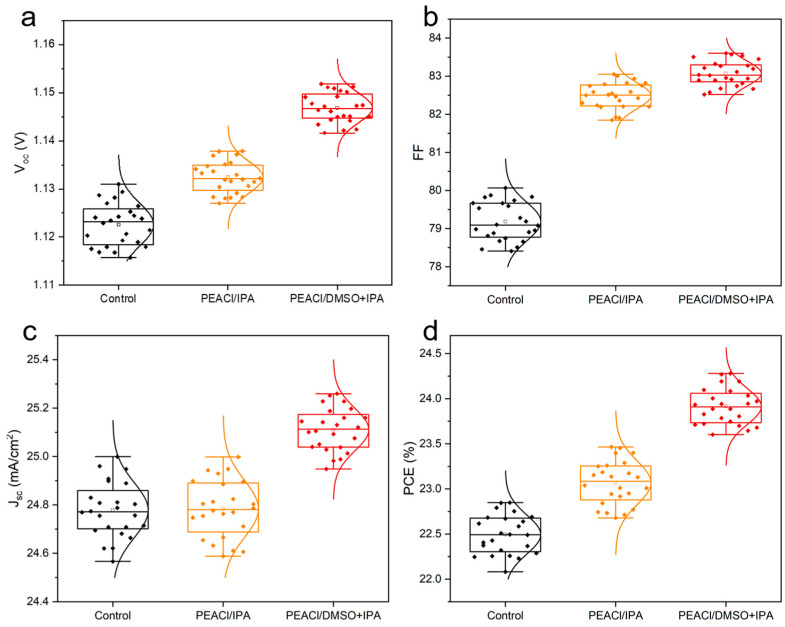
Statistical box plots for (**a**) V_OC_, (**b**) FF (**c**) J_SC_, and (**d**) PCE.

**Figure 10:**
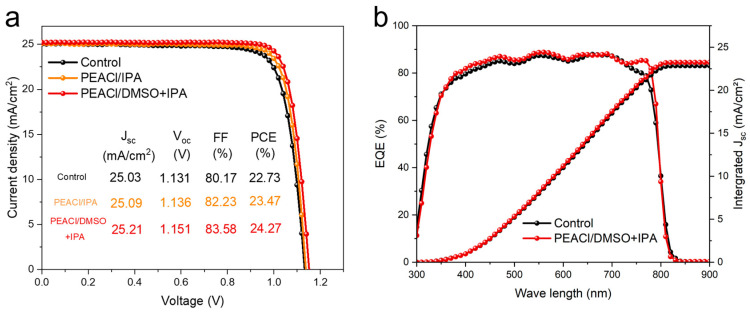
(**a**) J-V curves of different devices with or without PEACl treatment. (**b**) EQE spectra.

To evaluate the photovoltaic performance of perovskite solar cells after modification with PEACl, we fabricated hybrid perovskite solar cells with a p-i-n structure of FTO/HTL/perovskite/PEACl/C_60_/BCP/Ag. Passivation with PEACl confirmed the presence of fewer defects, high charge extraction and transport, lower non-radiative recombination, and a denser and more uniform passivation layer. In the box plot in Figure 9, we present the photovoltaic parameters of 24 individual devices, and we can see that V_oc_ and FF were significantly improved. (The optimal concentration of PEACl and the experimental results of the comparison of PEAI and PEACl are shown in Figures S1–S3), moreover, the device based on mixed solvent passivation remained the best-performing one, achieving an efficiency of 24.27%. In contrast, the highest power conversion efficiencies (PCEs) of the control and single-solvent passivation groups were 22.73% and 23.47%, respectively. The *J-V* curves for each type are shown in Figure 10a.

External quantum efficiency (EQE) measurements were performed to verify the measured Jsc, as shown in Figure 10b. By integrating the incident photon-to-current conversion efficiency, integrated Jsc values of 22.61 and 23.34 mA·cm^−2^ for the control and target devices were obtained, respectively. This matches the measured Jsc values from the J-V curves. The EQE of the target device exhibits a slight increase over the entire visible-light region, which is due to the improved film quality and efficient charge transport.

**Figure 11:**
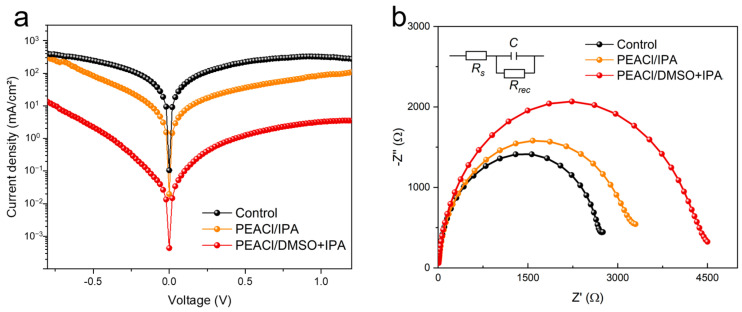
(**a**) Dark *J–V* curves. (**b**) The Nyquist plots of control devices and devices with PEACl treatment, recorded at 1.1 V in dark conditions.

To deeply analyze the suppression mechanism of trap-assisted recombination, a dark current–voltage (J-V) characteristic test was performed. The test results (Figure 11a) show that the dark current density of the optimized device exhibited a significant downward trend compared to that of control group. This phenomenon directly reflects a significant reduction in the bulk defect concentration, which is of great significance for improving the Voc of the device [29,36,37].

The devices treated with different solvent systems were further analyzed by electrochemical impedance spectroscopy (EIS). The Nyquist diagram obtained at a 1.1 V dark bias (Figure 11b) showed that compared to the control group, the devices treated with PEACl showed a significant increase in composite resistance (Rrec) and a significant decrease in series resistance (Rs). Among them, the performance of the device treated with the binary mixed-solvent system was the most prominent, with the Rrec value reaching the highest level and the Rs value decreasing to the lowest. This optimization of the electrical properties confirms the effective reduction in interface defect-assisted trap states, which not only promotes the efficient transport of carriers, but also inhibits the non-radiative recombination process [38].

**Figure 12:**
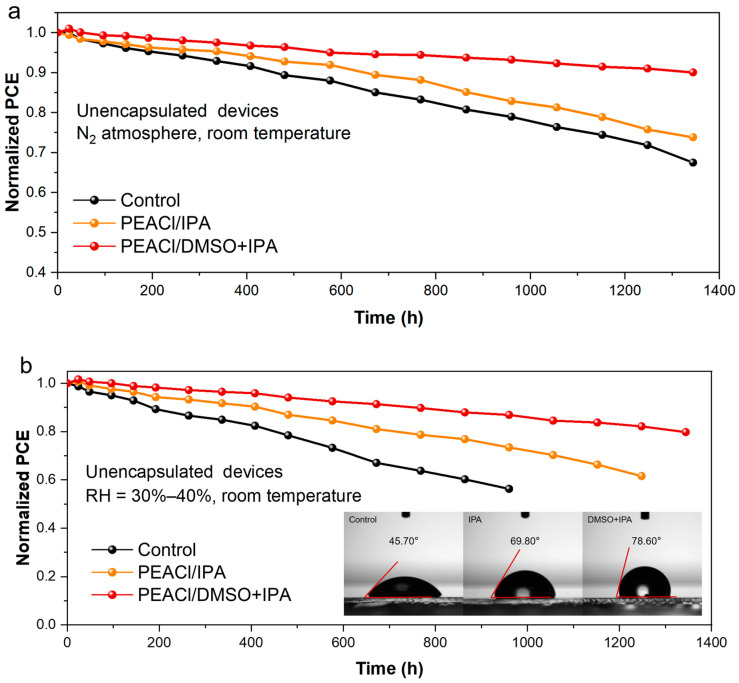
Stability of unencapsulated PSCs. (**a**) Stored in an N_2_ atmosphere at room temperature. (**b**) In RH = 30–40%, room temperature; the inset figures show the contact angle of PEACl-treated PSCs.

The final experiment focused on the regulation mechanism of the PEACl treatment process on the stability of the unencapsulated devices. After 1344 h of aging in a nitrogen atmosphere, the device treated with the binary mixed solvent maintained approximately 90% of the initial efficiency, while the efficiency retention rates of the control group and the single-IPA-solvent treatment group were 67% and 73%, respectively (Figure 12a). The surface contact angle test showed that the contact angles of the control, single-solvent, and binary mixed-solvent treatment groups were 45.7°, 69.8°, and 78.6°, respectively, indicating that PEACl treatment significantly improved the hydrophobic properties of the perovskite material, thereby improving its moisture resistance. Notably, in the binary solvent system, PEACl can react more fully with the perovskite film to form a denser passivation layer structure. The stability test results in an environment with a relative humidity of 30–40% showed that the device treated with the binary solvent maintained an initial efficiency of 80% after 1344 h of aging (Figure 12b).

## 4. Conclusions

In summary, this study demonstrates that co-solvent engineering is a pivotal lever for optimizing PEACl passivation in perovskite solar cells. Three key advances are achieved as follows:

(1) **Synergistic solvent dynamics:** The DMSO: IPA (1:100) mixed solvent establishes a dynamic equilibrium, where DMSO retards the crystallization process, promotes the growth of large grains, and improves the uniformity of thin films through strong coordination, while IPA’s rapid volatilization confines 2D perovskite growth to an ultrathin, pinhole-free morphology. This dual mechanism reduces interfacial defect density by 51% compared to the control.
(2) **Efficiency–stability co-optimization:** The optimized device achieves a PCE of 24.27% (V_OC_ = 1.15 V, FF = 83.5%) and retains 90% of its initial efficiency after 1344 h in inert atmospheres, addressing the perennial efficiency–stability trade-off.
(3) **Universal design principles:** The proposed “solvent coordination-volatilization kinetics” model transcends PEACl-based systems, offering a blueprint for tailoring passivation layers in diverse perovskite compositions. This model hinges on two transferable parameters: (a) coordination strength—solvents with Lewis base functionality (e.g., DMSO and dimethylformamide) can tune passivator–substrate bonding to align molecular orientation, and (b) volatilization gradient—low-boiling-point solvents (e.g., IPA and ethyl acetate) regulate phase separation kinetics, preventing excessive 2D phase growth. For instance, substituting PEA^+^ with bulkier ammonium cations (e.g., butylammonium) would require adjusting the DMSO/IPA ratio to balance steric effects and crystallization dynamics. Similarly, replacing Pb^2+^ with Sn^2+^ in lead-free perovskites could leverage alternative coordination motifs (e.g., Sn-S bonds with thiourea-based solvents).

This framework is experimentally validated through in situ XRD (suppressed PbI_2_ at 12°–13.4°) and TRPL (carrier lifetime extended to 412 ns), which correlate solvent properties with passivation quality. Future work could extend this model to large-area blade coating or slot-die printing, where solvent engineering critically governs film uniformity. Computational studies (e.g., density functional theory on solvent-Pb^2+^ binding energies) may further refine coordination–volatility design rules.

## Data Availability

Dataset is available on request from the authors.

## Supplementary Materials

The following supporting information can be downloaded at: https://www.mdpi.com/article/10.3390/nano15090699/s1, Figure S1: Statistical boxplots of (a) V_OC_, (b) FF, (c) J_SC_, and (d) PCE for devices under different concentrations of PEACl passivation; Figure S2: Statistical boxplots of (a) V_OC_, (b) FF, (c) JSC, and (d) PCE of devices with 1mg/mL PEACl passivation at different ratios of DMSO:IPA passivation; Figure S3: Statistical boxplots of (a) V_OC_, (b) FF, (c) J_SC_, and (d) PCE for devices at the same concentration of PEAI and PEACl passivation.
