# Supplementary material for: Solvent-Engineered PEACl Passivation: A Pathway to 24.27% Efficiency and Industrially Scalable Perovskite Solar Cells
Source: Nanomaterials (Basel). 2025 May 6;15(9):699. doi: 10.3390/nano15090699 (PMC12073452; doi:10.3390/nano15090699)
Supplement: Supplementary file 1 [file nanomaterials-15-00699-s001.zip › nanomaterials-3570304-supplementary.pdf]

SUPPORTING INFORMATION

Article

Solvent-Engineered PEACl Passivation: A Pathway to 24.27% Efficiency and Industrially Scalable Perovskite Solar Cells

Min Xin[1,2], Ihtesham Ghani[2], Yu Zhang[2], Huaxi Gao[1,2], Danish Khan[2], Xin Yang[1,*] Zeguo Tang[2,*]

[1] School of energy and Environmental Sciences, Yunnan Normal University,Juxian Road 768, Chenggong, Kunming 650500, China
[2] The College of New Materials and New Energies, Shenzhen Technology University, Lantian Road 3002, Pingshan, Shenzhen 518118, China

* Correspondence: yangxinzju@zju.edu.cn (X. Yang); tangzeguo@sztu.edu.cn (Z. Tang)

To verify the optimal concentration of PEACl, we measured the optoelectronic parameters of 24 individual devices for PEACl passivation at 0.5 mg/mL, 1 mg/mL, and 1.5 mg/mL. To verify the optimal ratio of DMSO and IPA, we measured the optoelectronic parameters of 24 individual devices for PEACl passivation of 1:50, 1:100, and 1:200. The results showed that the device achieved the highest PCE at a PEACl concentration of 1 mg/mL and a DMSO:IPA of 1:100.

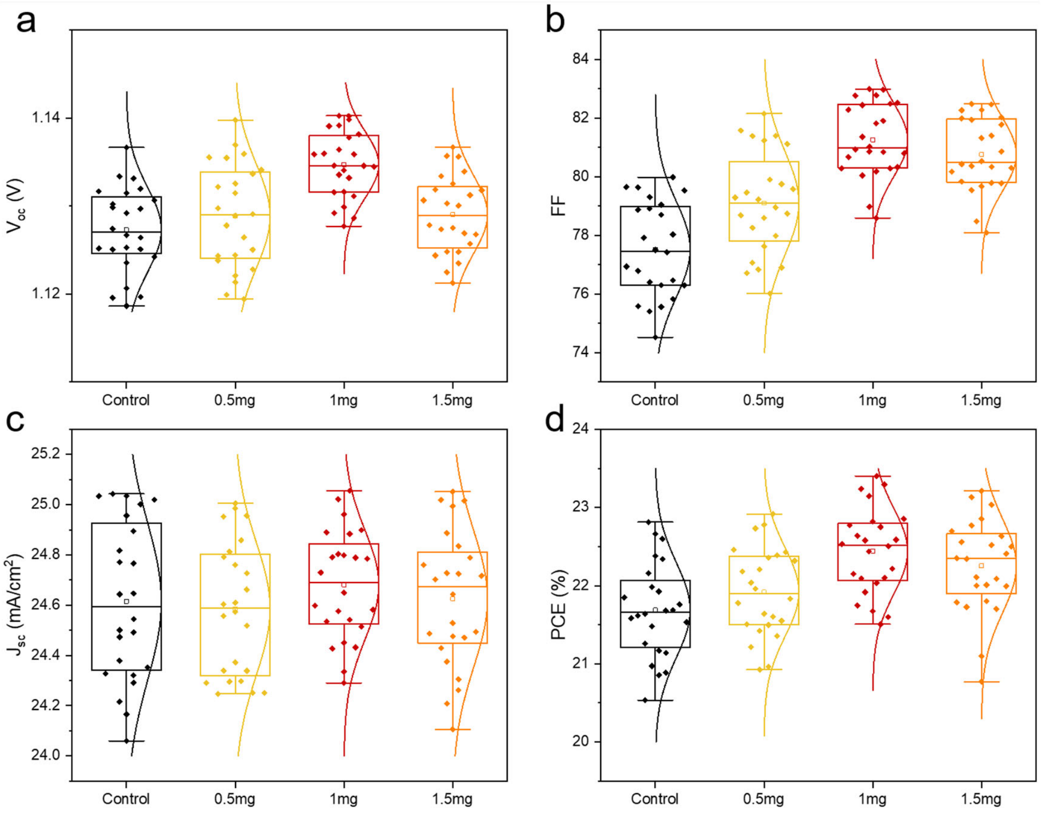

Figure S1. Statistical boxplots of (a) V_{OC}, (b) FF, (c) J_{SC}, and (d) PCE for devices under different concentrations of PEACl passivation.

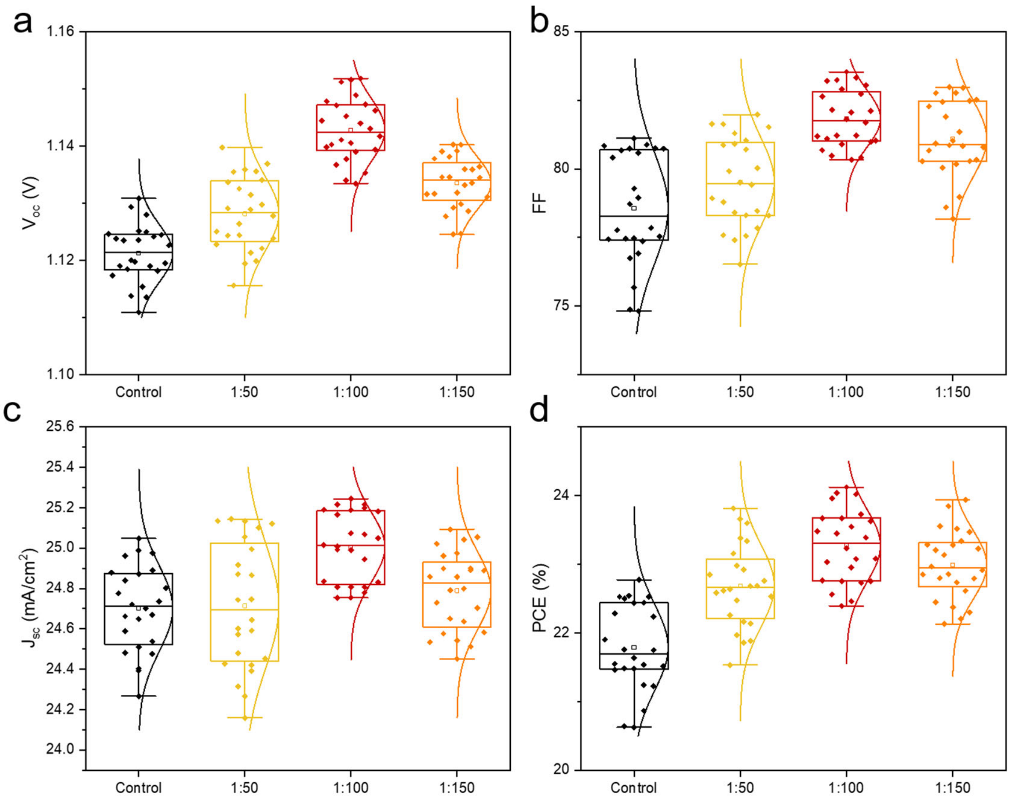

Figure S2. Statistical boxplots of (a) V_{OC}, (b) FF, (c) J_{SC}, and (d) PCE of devices with 1mg/mL PEACl passivation at different ratios of DMSO:IPA passivation.

Comparative experiments between PEAI and PEACl were systematically conducted in our preliminary studies. The statistical results, derived from twenty-four individual devices per test group, revealed that PEACl demonstrated markedly superior performance enhancement compared to PEAI. This disparity can be primarily attributed to the more favorable energy band alignment within the 2D/3D heterojunction structure formed by PEACl-modified perovskite films. The results are shown in Figure 3.

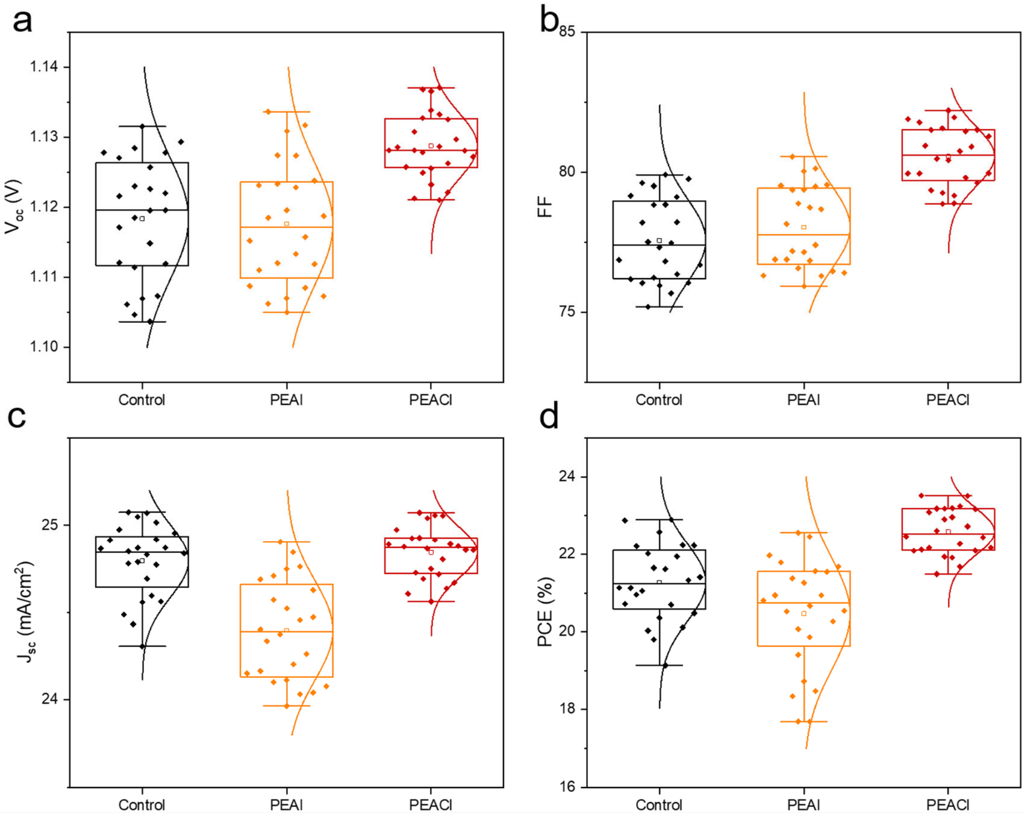

Figure S3. Statistical boxplots of (a) V_{OC}, (b) FF, (c) J_{SC}, and (d) PCE for devices at the same concentration of PEAI and PEACl passivation.
